# Storage Artifact Masquerading as Yeast: Presenting a Diagnostic Pitfall

**DOI:** 10.1155/2022/3326214

**Published:** 2022-10-08

**Authors:** Elena M. Fenu, Tawfeq Naal, Elizabeth Palavecino

**Affiliations:** Department of Pathology, Wake Forest Baptist Medical Center, Winston-Salem, NC, USA

## Abstract

Abnormal cell morphology can result from prolonged specimen storage, both for red and white blood cells. In particular, nuclear pyknosis of segmented neutrophils can occur in both peripheral blood and body fluids and may represent a diagnostic pitfall as it can mimic intracellular yeast or bacteria morphology. Pathologists are frequently asked to examine body fluid smears which are thought to contain microorganisms, and the presence of an unexpected organism can be especially concerning. Morphologic changes from prolonged storage may be encountered infrequently, and it is important to be aware of them in order to avoid misrepresentation, as additional work-up may be required for a suspected case of an unexpected fungal infection.

## 1. Case Report

A 32-year-old woman presented to the emergency department with fever (100.6 F), chest pain, and shortness of breath. She had a history of mixed connective tissue disease with features of systemic lupus erythematosus, systemic sclerosis, and Sjögren's and was on chronic immunosuppression with prednisone, mycophenolic acid (Myfortic), and hydroxychloroquine (Plaquenil). Upon admission, she was found to have an elevated C-reactive protein and erythrocyte sedimentation rate and a pericardial effusion, consistent with pericarditis. She was treated with colchicine and ibuprofen, however, a follow-up echocardiogram showed worsening of the pericardial effusion with signs concerning for tamponade, and a therapeutic pericardiocentesis was performed.

**Figure 1:**
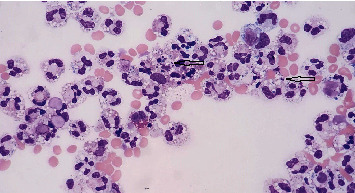
Pericardial fluid Giemsa stain at 400× magnification, arrows show variably sized round, fragmented elements with “budding.”

The pericardial fluid specimen was collected at 17 : 43 and, following review by a pathology resident, the final cell count result was finalized at 10 : 33 the following morning. The pericardial fluid cell count with differential showed 759 polynuclear cells/mm^2^, as well as intracellular forms morphologically suspicious for yeasts (Figure 1). A culture was ordered. The initial Gram stain showed 1+ white blood cells with no organisms seen, and both bacterial and fungal cultures for this specimen showed no growth. The pericardial fluid cytology specimen showed marked acute inflammation with no malignant cells, and a Grocott' s methenamine Silver (GMS) stain failed to highlight any fungal organisms. *Histoplasma* and *Blastomyces* urine antigens were both negative. Upon review of the cell count slide with the attending microbiologist, it was determined that the possible “yeast forms” represented nuclear pyknosis and not a true fungal infection. The patient subsequently recovered from her pericardiocentesis, her symptoms improved, and she was discharged home.

## 2. Discussion

Fungal pericarditis is extremely rare, with only a handful of cases reported in literature. Affected patients are usually immunosuppressed, and many have a history of recent cardiothoracic surgery, esophageal surgery, or solid organ transplant [1]. For example, *Candida* pericarditis was identified in a patient with an esophagopericardial fistula [2]. *Cryptococcus neoformans* pericarditis was identified in a bilateral lung transplant recipient and resulted in a pericardial effusion with tamponade [3]. *Trichosporon beigelii* was cultured from pericardial and mediastinal fluid in a patient who had an intraaortic balloon pump inserted in preparation for cardiac transplantation [4]. Early diagnosis is critical as patients may be affected by cardiac tamponade and can have poor outcomes in spite of combined surgical drainage and antifungal therapy. As described above, the patient presented here did not have risk factor for fungal pericarditis, except for steroid use, and the clinical findings did not correlate with an infectious process.

There are many more documented cases of preanalytical issues affecting interpretation of peripheral blood smears or effusion cytology. For effusions and body cavity fluids in particular, proper handling and preparation is essential for successful interpretation of cytology specimens and cell counts [5]. Samples which cannot be evaluated immediately should be stored at 4°C for optimal preservation of cell morphology, immunophenotypic profile, and amplifiable DNA [6].

On a peripheral smear, crenation of erythrocytes is common, and platelet clumping with resulting pseudothrombocytopenia has been associated with blood stored in ethylenediaminetetraacetic acid (EDTA) [7]. Drying artifact or improper staining may simulate also pathologic red blood cell inclusions such as basophilic stippling [8]. On both peripheral smears and body fluid cell counts, white blood cells can undergo degenerative changes, particularly nuclear pyknosis. The nuclei of segmented neutrophils can show chromatin condensation and effacement of the strands separating the nuclear lobes which can mimic intracellular yeast forms. These nuclear changes have been documented in the white blood cells of both humans and animals after prolonged storage [9].

Unlike true intracellular yeasts, degenerated nuclear lobes of neutrophils will display size differences between the lobes, fine strands of chromatin connecting the nuclear lobes rather than true budding and associated karyorrhexis and apoptotic bodies. Basophilia and vacuolation of the cytoplasm of white blood cells, along with cytoplasmic blebbing, are also seen in prolonged storage and can be a clue that a cell's nuclear changes represent an artifact [10]. The species which have previously been implicated in fungal pericarditis will also not show purely intracellular forms. A careful morphologic analysis, along with correlation with additional clinical tests, such as fungal serologic studies and a Gram stain, can help prevent misdiagnosis due to this underrecognized diagnostic pitfall.

### 2.1. Take-Home Messages

- Cellular degeneration from prolonged storage may lead to nuclear pyknosis which can mimic intracellular yeast forms
- Careful examination of size and morphology of these nuclear remnants, together with the results of a Gram stain, can help distinguish between artifact and a true fungal infection

## Data Availability

Data sharing not applicable to this article as no datasets were generated or analyzed during the current study.
